# Putting a “C_60_ Ball” and Chain to Chlorin e6 Improves Its Cellular Uptake and Photodynamic Performances

**DOI:** 10.3390/ph16091329

**Published:** 2023-09-20

**Authors:** Manuele Di Sante, Alena Kaltenbrunner, Marco Lombardo, Alberto Danielli, Paolo Emidio Costantini, Matteo Di Giosia, Matteo Calvaresi

**Affiliations:** 1 Dipartimento di Chimica “Giacomo Ciamician”, Alma Mater Studiorum—Università di Bologna, Via Francesco Selmi 2, 40126 Bologna, Italy; manuele.disante2@unibo.it (M.D.S.); marco.lombardo@unibo.it (M.L.); 2 Dipartimento di Farmacia e Biotecnologie, Alma Mater Studiorum—Università di Bologna, Via Francesco Selmi 3, 40126 Bologna, Italy; alena.kaltenbrunner2@unibo.it (A.K.); alberto.danielli@unibo.it (A.D.)

**Keywords:** Chlorin e6 (Ce6), C_60_, photodynamic therapy (PDT), antenna system, phototheranostics

## Abstract

Chlorin e6 (Ce6) and fullerene (C_60_) are among the most used photosensitizers (PSs) for photodynamic therapy (PDT). Through the combination of the chemical and photophysical properties of Ce6 and C_60_, in principle, we can obtain an “ideal” photosensitizer that is able to bypass the limitations of the two molecules alone, i.e., the low cellular uptake of Ce6 and the scarce solubility and absorption in the red region of the C_60_. Here, we synthesized and characterized a Ce6–C_60_ dyad. The UV-Vis spectrum of the dyad showed the typical absorption bands of both fullerene and Ce6, while a quenching of Ce6 fluorescence was observed. This behavior is typical in the formation of a fullerene–antenna system and is due to the intramolecular energy, or electron transfer from the antenna (Ce6) to the fullerene. Consequently, the Ce6–C_60_ dyad showed an enhancement in the generation of reactive oxygen species (ROS). Flow cytometry measurements demonstrated how the uptake of the Ce6 was strongly improved by the conjugation with C_60_. The Ce6–C_60_ dyad exhibited in A431 cancer cells low dark toxicity and a higher PDT efficacy than Ce6 alone, due to the enhancement of the uptake and the improvement of ROS generation.

## 1. Introduction

An ideal photosensitizer [1,2] must have both photophysical and drug-like properties, including: (i) a high quantum yield of the intersystem crossing process, (ii) the generation of ROS with both type I and type II mechanisms, (iii) intense absorption in the “tissue optical window” (from 650 to 1350 nm), (iv) resistance to photodegradation, (v) accumulation in cancer cells, (vi) negligible dark toxicity, and (vii) high solubility and stability in physiological environments.

Chlorin e6 (Ce6) [3,4,5] and fullerene (C_60_) [6,7,8,9,10,11,12,13,14,15] are among the most known photosensitizers (PSs) for photodynamic therapy (PDT). On one side, Ce6 possesses a significant absorption in the red region of the visible spectrum and an intense fluorescence that may be used to develop theranostic applications [3,4,5]. However, due to its negative charges, Ce6 shows a poor cellular uptake, which determines a low accumulation in cancer cells [3,4,5]. The penetration of a photosensitizer into the cell is a crucial step to perform an efficient PDT treatment. To bypass this drawback, many Ce6 derivatives [3,4,5,16,17,18,19,20] and ad hoc delivery systems [3,4,5,21,22,23,24,25,26,27] were developed, aiming to improve Ce6 accumulation in cancer cells through passive and active targeting.

On the other side, fullerene C_60_ follows both the photophysical mechanisms (type I and II mechanisms) involved in the production of ROS, is highly resistant to photobleaching [6,7,8,9], and exhibits a strong tendency to pass the cellular membrane (it is commonly used as a delivery system [28,29,30]). However, the extremely low solubility of C_60_, even in organic solvents, and its poor absorption in the visible region of the spectrum limit its application in PDT [6,7,8,9]. Chemical functionalization of the fullerene cage can improve its solubility, while the use of “antenna systems” can enhance its photodynamic activity in the visible region [31,32,33,34,35,36,37,38]. The molecular antenna (donor) acts as a light collector, absorbing the light in the desired visible spectral region and then transferring the excitation energy or electrons to the fullerene (acceptor) [31,32,33,34,35,36,37,38].

Both of the PSs are characterized by a high quantum yield of the intersystem crossing process.

The combination of the chemical and photophysical properties of Ce6 and C_60_ could be a suitable strategy to obtain, in principle, an “ideal” photosensitizer that is able to bypass the limitations of the two molecules alone, including (i) poor cellular uptake for Ce6 and (ii) the lack of solubility in physiological environments and low absorption in the visible spectrum of C_60_ (chlorophylls are commonly used by nature as antenna molecules, and Ce6 is a chlorophyll-like donor).

Here, a novel procedure for the synthesis of a Ce6–C_60_ dyad is presented. The Ce6–C_60_ dyad was characterized from a chemical and photophysical point of view. The ability to generate ROS in physiological environments was investigated. The cellular uptake and the performance of the dyad in PDT were determined in vitro.

## 2. Results and Discussion

### 2.1. Synthesis and Characterization of the Ce6–C_60_ Dyad

**Figure 1:**
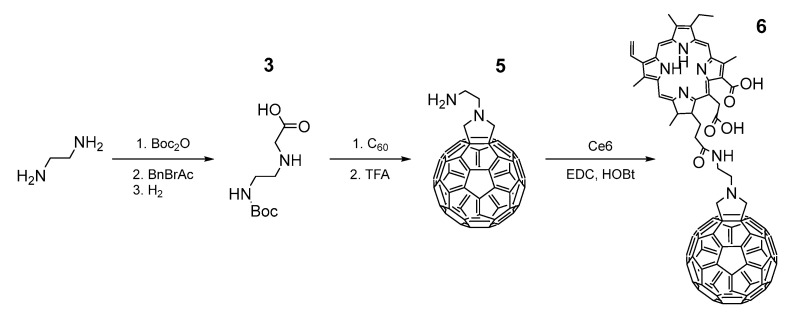
Synthesis of the Ce6–C_60_ (**6**) dyad.

The Ce6–C_60_ dyad was prepared via a coupling reaction between the carboxylic groups of the Ce6 molecule and a C_60_ derivative bearing an amine group (Figure 1). In brief, starting with ethylenediamine, a mono-protection with a Boc group was carried out, and the free amine was alkylated with benzyl bromoacetate. The deprotection of the carboxylic acid with a palladium on carbon mediated hydrogenation gave the α-amino acid **3**. A Prato reaction was carried out using **3** and formaldehyde to functionalize the C_60_. The Boc protecting group was removed through an acid hydrolysis, obtaining the fullerene amino derivative **5**.

An amidic coupling between **5** and Ce6 via EDC/HOBt activation gave the Ce6–C_60_ dyad (**6**). The mixture was purified using flash chromatography on silica gel, obtaining a dark-green to brownish solid.

**Figure 2:**
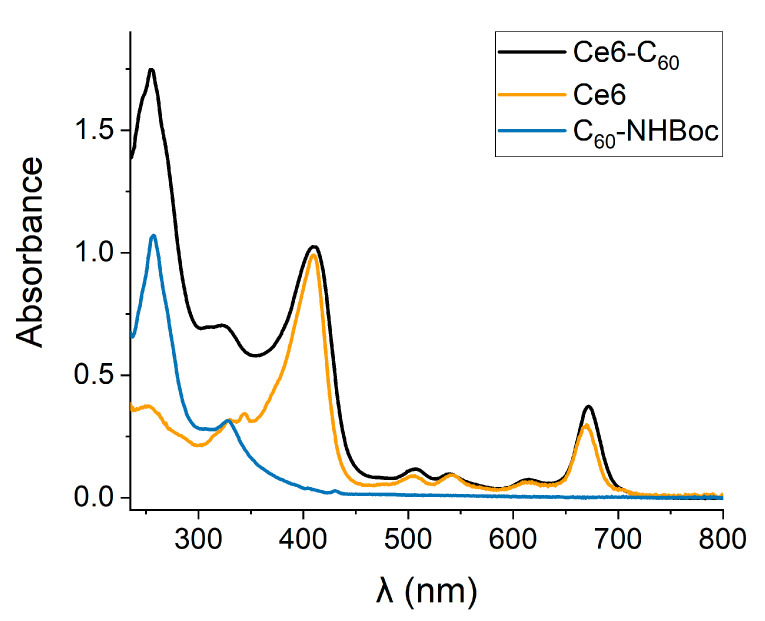
UV-Vis spectra of Ce6–C_60_ (black line), Ce6 (gold line), and C_60_-NHBoc (cyan line) in DCM.

Mass spectrometry (Figures S1 and S2) confirmed the covalent linkage of the dyad by the presence of a molecular peak with the expected isotopic pattern. The UV-Vis spectrum (Figure 2) of dyad **6** in DCM clearly showed both the diagnostic absorption bands of fullerene (256 nm and 324 nm) and Ce6 (406 and 668 nm).

Conjugation of the Ce6 with the fullerene derivative led to detectable changes in its absorption spectra; in particular, the red shift of the Q-band (3 nm in DCM, 5 nm in DMSO) and the broadening of the absorption bands were noticeable due to the interaction of Ce6 with the fullerene cage in the dyad, as was already reported for other fullerene–chlorin dyads [39].

**Figure 3:**
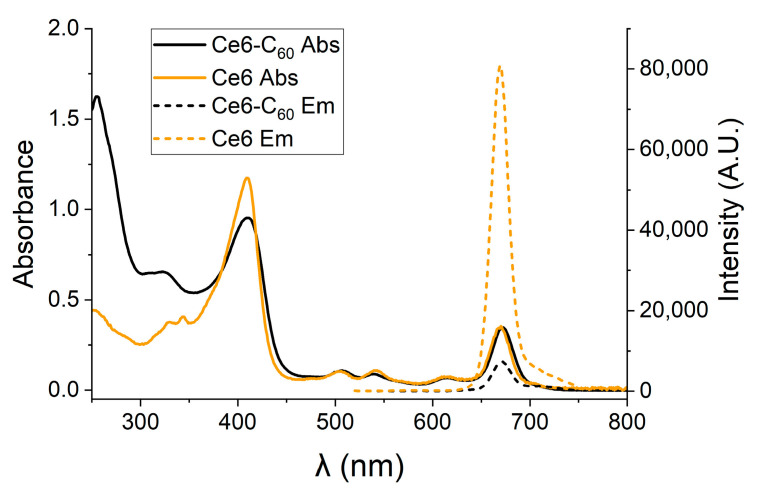
UV-Vis absorption spectra (solid lines) and emission spectra (dashed lines, λ_ex_ = 500 nm) of Ce6–C_60_ (black lines) and Ce6 (gold lines).

Fluorescence quenching of Ce6 by the fullerene moiety was observed. As reported in Figure 3, upon excitation of the Q-band (at 500 nm), the emission band of the Ce6-C_60_ dyad (centered at 670 nm) showed an intensity of about ten times lower (Φ_f_ = 0.015) than an isoabsorbing solution of Ce6 (Φ_f_ = 0.17) [40].

This behavior is typical for fullerene–dye dyads, and it is due to the intramolecular energy or electron transfer from the donor (Ce6) to the fullerene acceptor, competing with the radiative deactivation of the Ce6 singlet excited state. The residual fluorescence of the dyad was particularly interesting for theranostic applications.

### 2.2. Generation of ROS by the Ce6–C_60_ Dyad upon Red Light Irradiation

**Figure 4:**
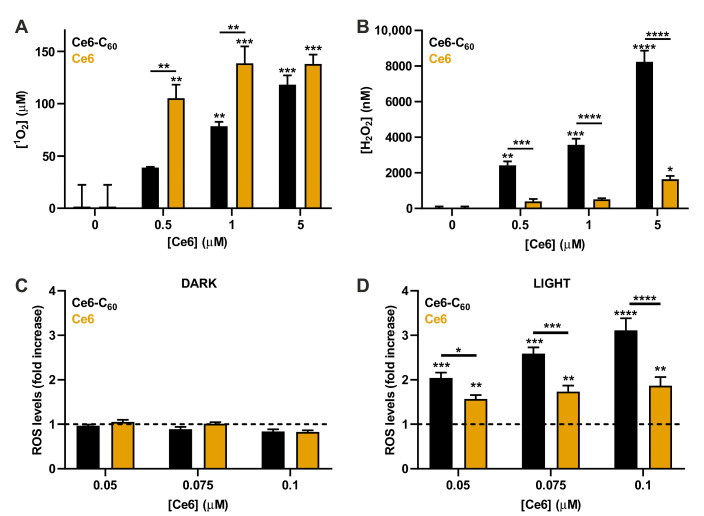
Quantification of ROS generated upon red light irradiation through (**A**) ABMDMA assay for the detection of ^1^O_2_ generated by the type II mechanism and (**B**) Amplex Red assay for the detection of H_2_O_2_ generated by the type I mechanism. Hydrogen peroxide levels were measured in cell cultures that were (**C**) kept in dark conditions or (**D**) irradiated with a red lamp through ROS-Glo^TM^ H_2_O_2_ assay. Statistical significance was calculated using one-way ANOVA followed by Dunnett’s multiple comparisons. Stars above the bars indicate significance in comparison to the untreated control (0 µM), while the significance between Ce6 and Ce6–C_60_ is indicated by stars above the line. * *p* < 0.05; ** *p* < 0.01; *** *p* < 0.001; **** *p* < 0.0001.

Ce6 is a well-known type II generator of ROS, characterized by the ability to produce singlet oxygen (^1^O_2_) upon red light irradiation. ABMDMA and Amplex Red assays, which determine, respectively, the generation of singlet oxygen (type II mechanism) and peroxides (type I mechanism), were used to investigate if the formation of the dyad affected the photosensitizing abilities of Ce6 upon red light irradiation. The Ce6–C_60_ dyad showed a reduced ability in ^1^O_2_ production (type II mechanism) compared to the Ce6 alone (Figure 4A). The singlet oxygen quantum yield, which was calculated by using the ABMDMA assay (Figure S3), was 0.32 for the Ce6–C_60_ dyad, which is about half of Ce6 alone (Φ_Δ_ = 0.60) [41,42]. On the other side, the production of peroxides (type I mechanism) was strongly improved, as expected from an antenna–fullerene system (Figure 4B) [38].

Intracellular production of ROS was also measured, confirming increased ROS production by the Ce6–C_60_ dyad compared to Ce6 after PDT treatment in the A431 cells (Figure 4C,D).

This feature is very intriguing from an application standpoint, as PSs that produce ROS through the type I mechanism are more and more preferred in anticancer PDT [43,44,45] because they can overcome the hypoxic environment present in tumor tissues. This is because they rely less on oxygen content.

### 2.3. Cellular Uptake of the Ce6–C_60_ Dyad

Ce6 bears three carboxylic side chains, and in physiological environments, it is negatively charged, limiting its cellular uptake. To improve the accumulation of Ce6 in cancer cells, two strategies are commonly pursued: (i) cationization of the Ce6 to improve the electrostatic interaction with the cellular membrane [46,47,48,49] and (ii) addition of a hydrophobic moiety at the macrocycle periphery, with a high affinity towards cellular membranes [46,47] enhancing its ability to penetrate membranes and enter cells.

To assess the uptake of Ce6 and the Ce6–C_60_ dyad, their internalization in A431 cells was quantitatively evaluated using flow cytometry analysis and recording the fluorescence signal of Ce6.

**Figure 5:**
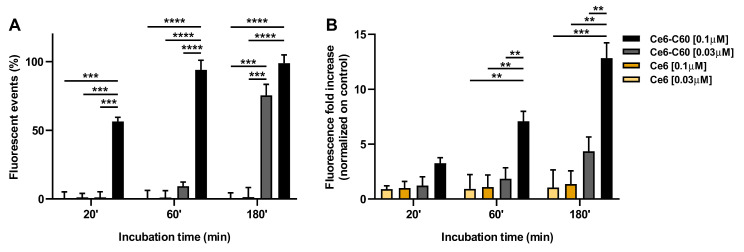
Cellular uptake of Ce6–C_60_ and Ce6 vs. incubation time. (**A**) % of fluorescent events in comparison to the control. (**B**) Fold increase in the fluorescence intensity normalized on the control. A431 cell line was incubated with equimolar concentrations of Ce6, in particular with 0.1 μM (grey bars for Ce6–C_60_ and yellow bars for Ce6) or 0.03 μM (black bars for Ce6–C_60_ and gold bars for Ce6). Statistical significance was calculated using one-way ANOVA followed by Dunnett’s multiple comparisons. ** *p* < 0.01; *** *p* < 0.001; **** *p* < 0.0001.

The data clearly show how the introduction of the hydrophobic fullerene moiety significantly improved the penetration of Ce6 through the cellular membrane, enhancing its uptake. In particular, after 3 h of incubation with 0.1 µM equimolar Ce6, a major difference was observed with a fluorescence increase of 12.8 ± 1.43 for Ce6–C_60_ and 1.36 ± 1.21 for Ce6 (Figure 5). In brief, putting a ball and chain to chlorin e6, such as C_60_, improves its cellular uptake.

### 2.4. Phototoxicity of the Ce6–C_60_ Dyad in A431 Cells Using Red Light Irradiation

**Figure 6:**
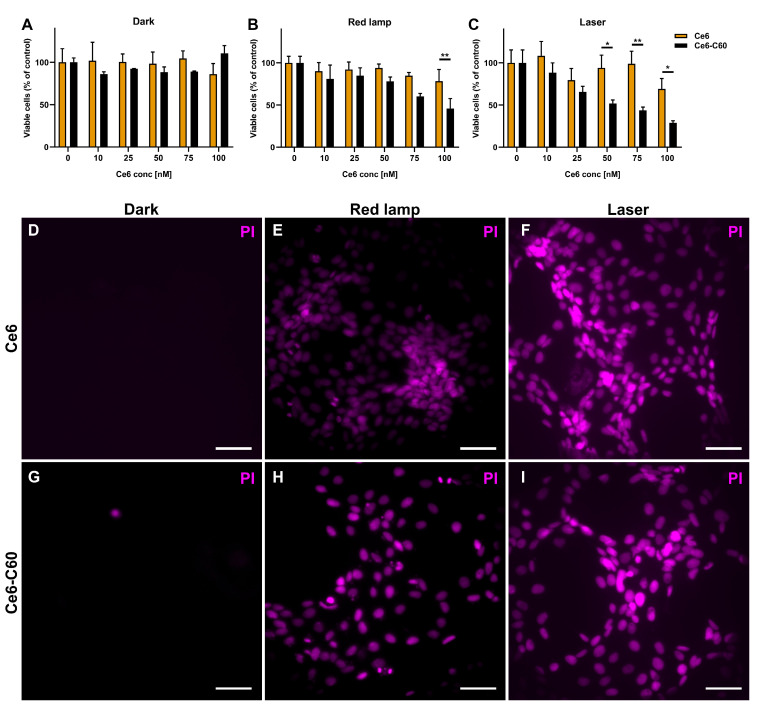
Cell viability of A431 cells after 24 h from PDT treatment with increasing concentrations of Ce6 (gold bars) or Ce6–C_60_ (black bars). After incubation with the sensitizers, cells were (**A**) kept in dark conditions (control), (**B**) irradiated with red lamp, or (**C**) irradiated with laser. Propidium iodide staining of A431 cells incubated with Ce6 (**D**–**F**) or Ce6–C_60_ (**G**–**I**) and kept in dark conditions (**D**,**G**), irradiated with red lamp (**E**,**H**), or irradiated with laser (**F**,**I**). Scale bar = 50 µm. Statistical significance was calculated using one-way ANOVA followed by Dunnett’s multiple comparisons test or t-test. * *p* < 0.05; ** *p* < 0.01.

Considering the outcomes of the internalization experiments, we investigated the phototoxic activity of Ce6 and the Ce6–C_60_ dyad against A431 cells. No significant dark toxicity was observed for Ce6 or the Ce6–C_60_ dyad until a concentration of 0.1 μM was reached (Figure 6). This aspect is extremely interesting because the conjugation of Ce6 to C_60_ overcomes two significant restrictions on fullerene use in nanomedicine: (i) its lack of solubility in physiological environments and (ii) its low biocompatibility. Irradiation of the cells with a red lamp (45 min at 6.3 mW/cm^2^, see Figure S4 for the spectral profile of the lamp) or with a laser (660 nm, 1 min, 2.66 W/cm^2^) showed an enhanced killing ability of the Ce6–C_60_ dyad compared to Ce6 (Figure 6). The conjugation of C_60_ to Ce6 caused a significant reduction in the viability of the A431 cells in a dose-dependent manner. Cell death after PDT treatment was confirmed by PI staining of the A431 cells. In both red-lamp and laser-irradiated samples, the presence of numerous PI-labeled cell nuclei was evident, demonstrating the cellular damage induced by the PDT treatment, which caused membrane permeabilization. Conversely, no PI-positive cells were observed in the samples that were kept in the dark (Figure 6D–I). In line with the uptake studies that demonstrate a greater internalization of Ce6–C_60_, it appears that (i) the photokilling activity of the Ce6–C_60_ dyad occurs at a lower concentration than Ce6 alone, and (ii) the PDT efficiency of Ce6–C_60_ is approximately twice that of Ce6 (69.01% ± 12.30% for Ce6 and 28.82% ± 2.51% for Ce6–C_60_ after laser PDT treatment of samples incubated with the highest Ce6 concentration) (Figure 6).

## 3. Materials and Methods

### 3.1. Materials

Ethylenediamine (≥99.5%), Di-tert-butyl dicarbonate (Boc_2_O, ≥98.0%), Benzyl bromoacetate (96%), Triethylamine (TEA, ≥99%), Palladium on carbon (Pd/C, 10 wt.%), Fullerene-C_60_ (98%), *N*-(3-Dimethylaminopropyl)-*N*′-ethylcarbodiimide hydrochloride (EDC, ≥98.0%), 1-Hydroxybenzotriazole hydrate (HOBt, ≥97.0%), 9,10-anthracenediyl-bis(methylene)dimalonic acid (ABMDMA), 10-Acetyl-3,7-dihydroxyphenoxazine (Amplex Red), and Type VI-A Peroxidase from horseradish lyophilized powder (HRP) were purchased from Merck (Darmstadt, Germany). Trifluoroacetic Acid (TFA, ≥99%) was purchased from TCI (Tokyo, Japan). Chlorin e6 (Ce6, ≥90%) was purchased from Cayman Chemical (Ann Arbor, MI, USA). All the reagents were used without further purifications. Flash chromatography purifications were performed using Merck Geduran silica gel (40–63 μm particle size). Thin-layer chromatography analyses (TLC) were carried out by using Merck 60 F254 plates.

The ^1^H and ^13^C NMR spectra were recorded on a Varian INOVA 400 NMR instrument with a 5 mm probe. Chemical shifts (δ) were expressed in ppm and coupling constants (*J*) in Hertz (Hz).

HPLC-MS characterizations were carried out on an Agilent Technologies HP1260 instrument. A Phenomenex Gemini C18 3 μm (100 × 3 mm) column was used for the chromatographic separation, with mobile-phase H_2_O/CH_3_CN, gradient of CH_3_CN from 30% to 80% in 8 min, 80% CH_3_CN until 22 min, then up to 90% CH_3_CN in 2 min, flow rate of 0.4 mL/min. Mass spectrometric detection (ESI-MS) was performed in the full-scan mode from 50 to 3000 *m/z* using the following parameters: (i) scan time of 0.1 s, (ii) ESI spray voltage of 4000 V, (iii) nitrogen gas pressure of 30 psi, (iv) drying gas flow rate of 8.0 L/min, and (v) fragmentor voltage 30 V.

High-resolution MS (HR ESI-MS) characterizations were carried out with direct flow injection using a Xevo G2-XS ESI-QTOF mass spectrometer (Waters Corporation, Milford, Worcester, MA, USA). Mass spectrometric detection was performed in the full-scan mode from *m/z* 50 to 2000 using the following parameters: (i) scan time of 0.15 s in the positive ion mode with (ii) cone voltage of 30 V, (iii) capillary voltage of 3 kV, (iv) source temperature of 120 °C, (v) desolvation temperature of 600 °C, (vi) cone gas flow of 50 L/h, and (vii) desolvation gas flow of 1000 L/h. In the negative ion mode, the following parameters were used: (i) cone voltage of 45 V, (ii) capillary voltage of 2.5 kV, (iii) source temperature of 150 °C, (iv) desolvation temperature of 350 °C, (v) cone gas flow of 50 L/h, and (vi) desolvation gas flow of 800 L/h.

The absorption spectra were recorded using a Cary60 UV-Vis spectrophotometer (Agilent Technologies, Stockport, UK). The fluorescence spectra were recorded with an Edinburgh FLS920 equipped with a Hamamatsu R928P photomultiplier.

A LED SMD 50W RGB, SKU 5691, VT-4752 (V-TAC, Milan, Italy) was used as a red light source, with an irradiance of 6.32 mW/cm^2^ and an emission band centered at 627 nm (Figure S4).

A 660 nm CW fiber-coupled infrared diode laser (FC-660, CNI Optoelectronics, Changchun, China) was used for the in vitro PDT treatment. The 660 nm laser was characterized by an irradiance of 2.66 W/cm^2^. The multiwell plate was perpendicular to the top-down, perpendicular laser irradiation.

### 3.2. Synthesis of Fullerene Derivative and Ce6–C_60_ Dyad

**Scheme 1:**
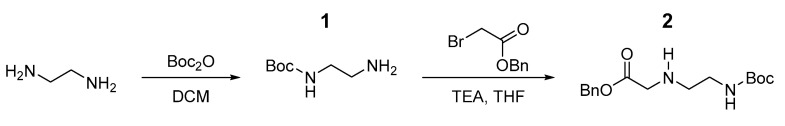
Synthesis of the intermediate **2**.

Ethylenediamine (1.00 mL, 12.0 mmol) was dissolved in anhydrous dichloromethane (DCM, 15 mL) under a nitrogen atmosphere, and di-*tert*-butyl dicarbonate (Boc_2_O, 873 mg, 4.00 mmol), which was previously dissolved in 5 mL of anhydrous DCM, was added dropwise at 0 °C (Scheme 1). After stirring at room temperature for 3 h, the mixture was checked by TLC (DCM:MeOH 9:1), and brine (20 mL) was added to the reaction solution. The extraction was carried out by using ethyl acetate (3 × 20 mL), and then the combined organic fractions were dried over anhydrous sodium sulfate, and the filtrate concentrated in the vacuum. The crude product, obtained as a pale-yellow oil, was used without further purification.

The crude mixture of **1** was dissolved in anhydrous tetrahydrofuran (THF, 8 mL) with triethylamine (TEA, 0.84 mL, 6 mmol) under a nitrogen atmosphere (Scheme 1). Lastly, benzyl bromoacetate (0.63 mL, 4 mmol) was added dropwise at 0 °C, observing the formation of a white solid. After stirring overnight at room temperature, water (20 mL) was added, and the mixture was extracted with ethyl acetate (3 × 20 mL), dried over anhydrous sodium sulfate, and the filtrate concentrated in vacuo. Column chromatography on silica gel (eluent: DCM:MeOH 98:2) gave the product **2** (666 mg, 2.16 mmol) as a yellowish oil in a 54% yield, as calculated over the two steps [50].

^1^H NMR (400 MHz, Chloroform-*d*) (Figure S5): δ 7.40–7.31 (m, 5H), 5.17 (s, 2H), 5.05 (broad s, 1H), 3.48 (s, 2H), 3.23 (q, *J* = 5.3 Hz, 2H), 2.78 (t, *J* = 5.7 Hz, 2H), 1.44 (s, 9H).

^13^C NMR (100 MHz, Chloroform-*d*) (Figure S6): δ 172.4, 156.0, 135.5, 128.6, 128.4, 128.3, 77.2, 66.6, 50.5, 48.7, 40.1, 28.4.

HPLC-MS (Acetonitrile) (Figure S7) Rt: 5.63 min, [M+H]^+^ 309.40.

**Scheme 2:**
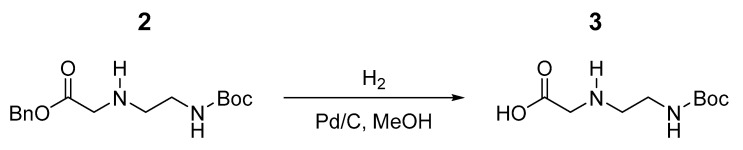
Synthesis of intermediate **3**.

Pd/C (10 wt. %, 106 mg, 0.10 mmol) was added to an anhydrous methanol solution (5 mL) of **2** (308 mg, 1 mmol), and the mixture was stirred overnight under a hydrogen atmosphere (Scheme 2). Celite filtration removed the catalyst, and then the solvent was evaporated. The product was washed with diethyl ether to give a white solid (176 mg, 0.81 mmol) in 81% yield.

^1^H NMR (400 MHz, CD_3_OD) (Figure S8): δ 3.49 (s, 2H), 3.35 (t, *J* = 5.9 Hz, 2H), 3.09 (t, *J* = 5.9 Hz, 2H), 1.43 (s, 9H).

^13^C NMR (100 MHz, CD_3_OD) (Figure S9): δ 170.7, 158.7, 80.8, 50.6, 48.9, 38.1, 28.7.

HPLC-MS (Acetonitrile) (Figure S10) Rt: 1.28 min, [M+H]^+^ 219.20.

**Scheme 3:**
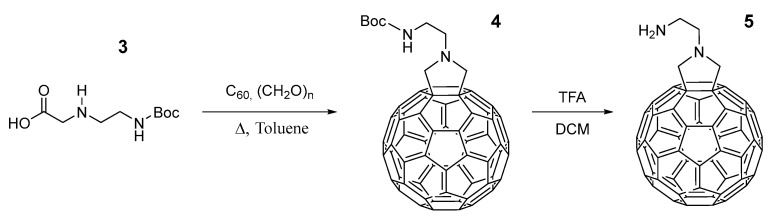
Synthesis of intermediate **5**.

A toluene solution (120 mL) of C_60_ (248 mg, 0.34 mmol), paraformaldehyde (21 mg, 0.69 mmol), and amino acid **3** (60 mg, 0.28 mmol) was heated to reflux for 1 h under a nitrogen atmosphere (Scheme 3). After cooling to room temperature and checking the formation of a brown spot with TLC (toluene:AcOEt 95:5), the product was purified on silica gel using column chromatography, first eluting it with toluene to recover the unreacted C_60_ and then with toluene:AcOEt 97:3 to separate the mono from the bis-adducts. The brown solid was thoroughly washed with diethyl ether to obtain a pure product (65 mg, 72 μmol) in a 26% yield [51].

^1^H NMR (400 MHz, Chloroform-*d*:CS_2_ 1:1) (Figure S11) δ 5.27 (broad s, 1H), 4.51 (s, 4H), 3.72 (q, *J* = 5.8 Hz, 2H), 3.30 (t, *J* = 6.0 Hz, 2H), 1.54 (s, 9H).

HR ESI-MS (MeOH) (Figures S12 and S13): [M+H]^+^ 907.1178, [M+Na]^+^ 929.0993.

UV-Vis (DCM) (Figure S14) λ_max_ 430 nm, 327 nm, 257 nm.

A solution of **4** (65 mg, 72 μmol) in DCM (1 mL) and trifluoroacetic acid (1 mL) was stirred at room temperature for 3 h (Scheme 3). The solvent and the acid in excess were removed in vacuo, obtaining the product as an ammonium salt (C_60_∙2TFA) and as a brown solid (70 mg, 68 μmol) in a 94% yield. 

ESI-MS (MeOH) (Figure S15): [M+H]^+^ 806.98.

UV-Vis (DMSO) (Figure S16): λ_max_ 433 nm, 325 nm.

**Scheme 4:**
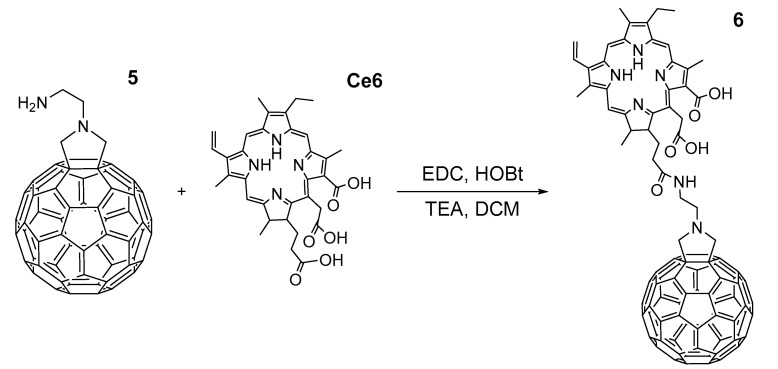
Amide coupling for the synthesis of the Ce6–C_60_ dyad.

Chlorin e6 (23 μmol, 14 mg), EDC∙HCl (46 μmol, 9 mg), and HOBt (46 μmol, 6 mg) were dissolved in anhydrous DCM (5 mL) and stirred at room temperature for 1 h. This solution was added dropwise to a suspension of the fullerene derivative **5** (23 μmol, 24 mg) and TEA (46 μmol, 6 μL) in anhydrous DCM (10 mL) (Scheme 4). The resulting reaction mixture was stirred overnight at room temperature under a nitrogen atmosphere. The solvent was then evaporated under reduced pressure, and the product was purified using flash chromatography on silica gel using 95:5 DCM/MeOH as eluent. Successive washing with diethyl ether (3 × 5 mL) gave the product as a dark-green to brownish solid (7 mg, 5 μmol) in a 21% yield.

HR ESI-MS (MeOH) (Figures S1 and S2): [M-H]^−^ 1383.3271, [M-CO_2_]^−^ 1339.3300, [M+OH]^−^ 1400.3185, [M+CH_3_O]^−^ 1414.3060.

UV-Vis (DCM): λ_max_ 672 nm, 615 nm, 539 nm, 506 nm, 409 nm, 322 nm, 255 nm.

^1^H NMR (400 MHz, Chloroform-*d*): δ 9.63 (s, 1H), 9.51 (s, 1H), 8.61 (s, 1H), 7.97 (dd, *J* = 17.8, 11.4 Hz, 1H), 6.26 (dd, *J* = 17.9, 1.6 Hz, 1H), 6.14 (dd, *J* = 11.5, 1.4 Hz, 1H), 4.44 (s, 4H), 4.15–4.05 (m, 2H), 4.04–3.93 (m, 2H), 3.80 (m, 6H), 3.36 (m, 3H), 3.26 (m, 6H), 3.19 (s, 1H), 2.62–2.52 (m, 2H), 2.35 (q, *J* = 7.6 Hz, 1H), 1.76–1.62 (m, 6H).

### 3.3. Detection of ROS Produced upon Red Light Irradiation

**Scheme 5:**
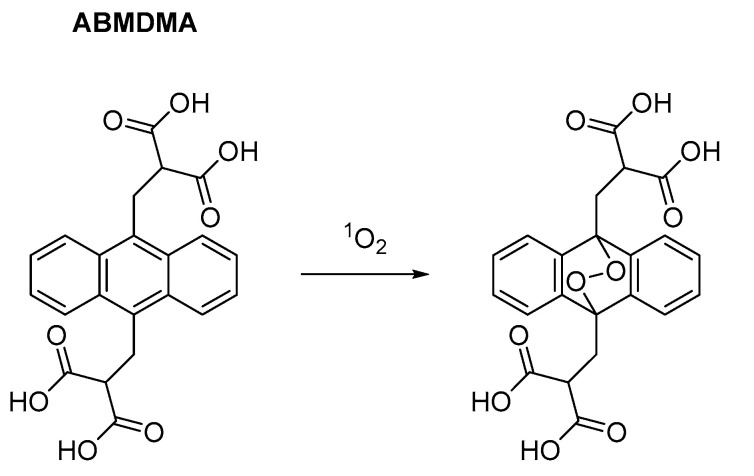
Reaction scheme of ABMDMA with singlet oxygen.

*ABMDMA assay.* 9,10-Anthracenediyl-bis(methylene)dimalonic acid (ABMDMA) was used as singlet oxygen detector. The disodium salt of ABMDMA reacts with ^1^O_2_ to give an endoperoxide (Scheme 5), which is detected as a decline of the absorbance at 401 nm [52,53].

DMSO stock solutions of Ce6 and Ce6–C_60_ were prepared with concentrations of 100, 20, and 10 μM, as determined from the Ce6 molar extinction coefficient at 670 nm. In a 96-well plate, 5 μL of these solutions were added to 95 μL of deuterated PBS (10 mM pH 7.4) in order to test the final concentrations of 5, 1, and 0.5 μM. Another well was filled with only deuterated PBS and DMSO (5%). Lastly, 3 μL of an ABMDMA 5 mM stock solution in DMSO was added to each well. The plate was irradiated for 1 h with a red light lamp.

The singlet oxygen quantum yield (Φ_Δ_) of Ce6 and Ce6–C_60_ was determined by irradiating the samples with the red lamp for up to 20 min and following the ABMDMA bleaching over time, with timesteps of 5 min. The Φ_Δ_ was calculated using the following equation:

(1) $$\Phi_{\Delta }^{Ce6-C_{60}}=\frac{k^{Ce6-C_{60}}}{k^{Ce6}}\times \Phi_{\Delta }^{\mathrm{Ce}6}$$

where *k* is the slope of the photooxidation rate of ABMDMA obtained by the curve-fitting equation (Figure S3). Absorbance data were collected before and after the irradiation using a PerkinElmer EnSpire^®^ Multimode Plate Reader (Cambridge, MA, USA).

**Scheme 6:**
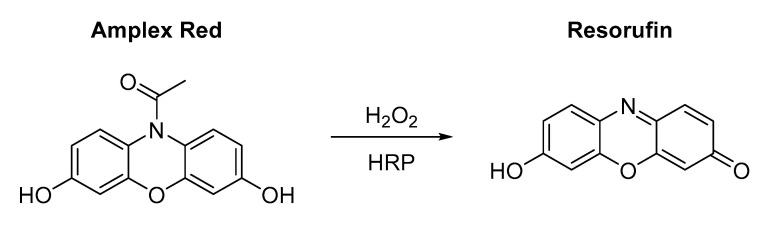
Reaction scheme of Amplex Red with hydrogen peroxide.

*Amplex Red assay.* The Amplex Red (AR) assay was used to evaluate the generation of peroxides upon irradiation with visible light. Colorless and nonfluorescent Amplex Red reacts with peroxides in a reaction catalyzed by horseradish peroxidase (HRP) to form Resorufin, which is a fluorescent molecule (Scheme 6) [10,54].

The concentration of the produced peroxides was calculated by measuring the fluorescence emission of Resorufin at 590 nm (excitation at 560 nm), subtracting the values of non-irradiated references that were kept in the dark. The fluorescence values were converted into hydrogen peroxide concentrations using a calibration curve obtained from standard solutions of H_2_O_2_. 10 μL of 50 mM AR stock solution in DMSO was diluted in 1 mL of 50 mM phosphate buffer at pH 7.4 (PB) to obtain a solution characterized by a final AR concentration of 500 μM. The final working solution (WS) was obtained by adding 10 μL of HRP at 0.4 mg/mL in PB to the AR solution. Stock solutions of Ce6 and Ce6–C_60_ in DMSO were prepared, with concentrations of 100, 20, and 10 μM. In a 96-well plate, 5 μL of these solutions was added to 85 μL of PB (final concentrations of Ce6 and Ce6–C_60_ of 5, 1, and 0.5 μM). The plate was irradiated with a red light lamp for 1 h and then 10 μL of the WS was added to each well. An equivalent plate was kept in the dark and then the WS was added. After 30 min, the fluorescence data were collected using a PerkinElmer EnSpire^®^ Multimode Plate Reader (Cambridge, MA, USA).

*Intracellular ROS Production.* Intracellular ROS production after PDT was determined by using ROS-Glo™ H_2_O_2_ Assay (Promega, Madison, WI, USA), following the protocol provided by the manufacturer. Briefly, around 25,000 A431 cells (ATCC CRL-1555) were incubated for 3 h with increasing concentrations of either Ce6 or Ce6–C_60_ prior to washing and irradiation with a red light lamp for 45 min. Immediately after the treatment, 20 µL of H_2_O_2_ substrate solution was added to each well (final volume 100 µL) and incubated for 20 min at 37 °C. A total of 100 µL of ROS-Glo™ detection solution was added to each sample, and the plate was incubated at room temperature for 20′. Relative luminescence was measured using EnSpire^®^ multimode plate reader (PerkinElmer, Cambridge, MA, USA).

### 3.4. Cellular Uptake

Cellular uptake of Ce6–C_60_ and Ce6 was evaluated on an A431 human epidermal carcinoma cell line (ATCC CRL-1555). A total of 100,000 cells were seeded in a 6-well plate and incubated overnight at 37 °C with 5% CO_2_. Next, cells were incubated with either 0.1 μM or 0.03 μM concentrations of Ce6–C_60_ or Ce6 for 20, 60, or 180 min. After the incubation, cells were washed twice with PBS to remove any unbound sensitizers. Subsequently, the cells were detached by trypsinization and analyzed using a citoFLEX S flow cytometer (Beckman Coulter, Brea, CA, USA). Data analysis was performed using CytExpert software 2.5 (Beckman Coulter) and FlowJo™ 10.9 software.

### 3.5. Cytotoxicity and Phototoxicity in A431 Cells

*MTT assay*. In a 96-well flat-bottom plate (Sarstedt, Germany), 25,000 cells of confluent growing A431 cells were seeded in 100 µL complete growth media RPMI (10% FBS, 1% L-Glutamine, 1% Penicillin-Streptomycin) per well and incubated overnight at 37 °C with 5% CO_2_. Cells were then incubated with increasing concentrations of Ce6 or Ce6–C_60_ (diluted in RPMI) for 3 h at 37 °C with 5% CO_2_. Afterwards, cells were washed twice with 1× PBS to remove any free sensitizer. For irradiation, 100 µL of 1× PBS was added per well, and the 96-well plate was irradiated for 45 min with a red lamp (6.3 mW/cm^2^, see Figure S4 for the spectral profile of the lamp) or a laser (660 nm, 2.66 W/cm^2^) for 1 min per well.

Cells were allowed to recover overnight in 200 µL complete RPMI per well at 37 °C with 5% CO_2_. The dark control plate was treated the same way but kept in the dark throughout. The cell viability was tested using MTT assay. Therefore, the cells were incubated in a final concentration of 0.5 mg/mL MTT (Sigma-Aldrich, St. Louis, MO, USA) in 100 µL RPMI per well for 90 min at 37 °C with 5% CO_2_. Next, the media were removed, and 100 µL per well of DMSO was added for the formazan solubilization. To determine the cell viability, the absorbance was measured at 570 and 690 nm wavelengths using the EnSpire multimode plate reader (PerkinElmer, Cambridge, MA, USA), and the absorbance at 570 nm was subtracted from the absorbance at 690 nm. The obtained values were normalized against a growth control that was not treated with sensitizer. The results are shown as the mean ± SD of three biological replicates, each with three technical replicates.

*Propidium iodide staining.* A431 cells were seeded on round coverslips placed inside a 6-well plate (Corning) and grown overnight at 37 °C with 5% CO_2_. Cells were then incubated in MTT assay, as described above, with either Ce6 or Ce6–C_60_ at a final Ce6 equimolar concentration of 0.1 μM and (i) kept in dark conditions, (ii) irradiated for 45 min with a red lamp (6.3 mW/cm^2^), or (iii) irradiated for 1 min with laser at 660 nm (2.66 W/cm^2^). Cells were then recovered for 24 h and stained for 30 min with Propidium iodide (PI) at a final concentration of 1 μM. A round coverslip was fitted into an Attofluor cell chamber (Invitrogen, Carlsbad, CA, USA) and covered with 1 mL of RPMI without phenol red complete (10% FBS, 1% PenStrep, and 1% L-glutamine). Cell images were then acquired with a Nikon Eclipse Ti-U inverted epifluorescence microscope.

## 4. Conclusions

In summary, we developed a novel phototheranostic platform for PDT treatment/fluorescence imaging based on the Ce6–C_60_ dyad. The Ce6–C_60_ dyad was synthesized via a coupling reaction between the carboxylic groups of the Ce6 molecule and a C_60_ derivative bearing an amine group.

Combining the peculiar properties of Ce6 and C_60_, the obtained Ce6–C_60_ dyad had the following characteristics: (i) efficient absorption in the red region of the visible spectrum; (ii) production of ROS following both type I and type II photophysical mechanisms; (iii) good biocompatibility and solubility in physiological environments; (iv) enhanced cellular uptake efficiency in vitro (with a fluorescence increase of 10.5 times after 3 h of incubation in comparison to Ce6); (v) simultaneous imaging capability and PDT performances. Remarkably, the dyad displayed superior photo-capabilities in terms of both peroxide generation and PDT efficiency on cancer cells. Notably, dyad irradiation resulted in the production of five times more peroxides and the photokilling of 40.2% more cancer cells compared to Ce6 at the same equimolar concentration.

This approach paves the way for the molecular engineering of highly efficient fullerene-based photosensitizers for anticancer PDT. The unique properties of the Ce6–C_60_ dyad suggest that, in perspective, it can be used as a new red light phototheranostic agent for fluorescence-imaging-guided PDT treatment.

## Data Availability

Data is contained within the article and supplementary material.

## Supplementary Materials

The following supporting information can be downloaded at https://www.mdpi.com/article/10.3390/ph16091329/s1, Figures S1 and S2: HR ESI-MS of compound **6**; Figure S3: Absorbance of ABMDMA vs. irradiation time; Figure S4: Spectral profile of the red light lamp; Figure S5: ^1^H NMR of compound **2**; Figure S6: ^13^C NMR of compound **2**; Figure S7: HPLC-MS of compound **2**; Figure S8: ^1^H NMR of compound **3**; Figure S9: ^13^C NMR of compound **3**; Figure S10: HPLC-MS of compound **3**; Figure S11: ^1^H NMR of compound **4**; Figure S12: HR ESI-MS of compound **4**; Figure S13: HR ESI-MS of compound **4**; Figure S14: UV-Vis spectrum of compound **4**; Figure S15: ESI-MS of compound **5**; Figure S16: UV-Vis spectrum of compound **5**.
