# Supplementary material for: Putting a “C60 Ball” and Chain to Chlorin e6 Improves Its Cellular Uptake and Photodynamic Performances
Source: Pharmaceuticals (Basel). 2023 Sep 20;16(9):1329. doi: 10.3390/ph16091329 (PMC10538216; doi:10.3390/ph16091329)
Supplement: Supplementary file 1 [file pharmaceuticals-16-01329-s001.zip › pharmaceuticals-2556708-supplementary.pdf]

Article

Putting a "C_{60} ball" and Chain to Chlorin e6 Improves Its Cellular Uptake and Photodynamic Performances

Manuele Di Sante,[1,†] Alena Kaltenbrunner, [2,†] Marco Lombardo,[1] Alberto Danielli,[2] Paolo Emidio Costantini,[2,*] Matteo Di Giosia,[1,*] Matteo Calvaresi[1,*]

[1] Dipartimento di Chimica "Giacomo Ciamician", Alma Mater Studiorum—Università di Bologna, Via Francesco Selmi 2, 40126 Bologna, Italy
[2] Dipartimento di Farmacia e Biotecnologie, Alma Mater Studiorum—Università di Bologna, Via Francesco Selmi 3, 40126 Bologna, Italy
* Correspondence: paolo.costantini4@unibo.it (P.E.C.); matteo.digiosia2@unibo.it (M.D.G.); matteo.calvaresi3@unibo.it (M.C.)

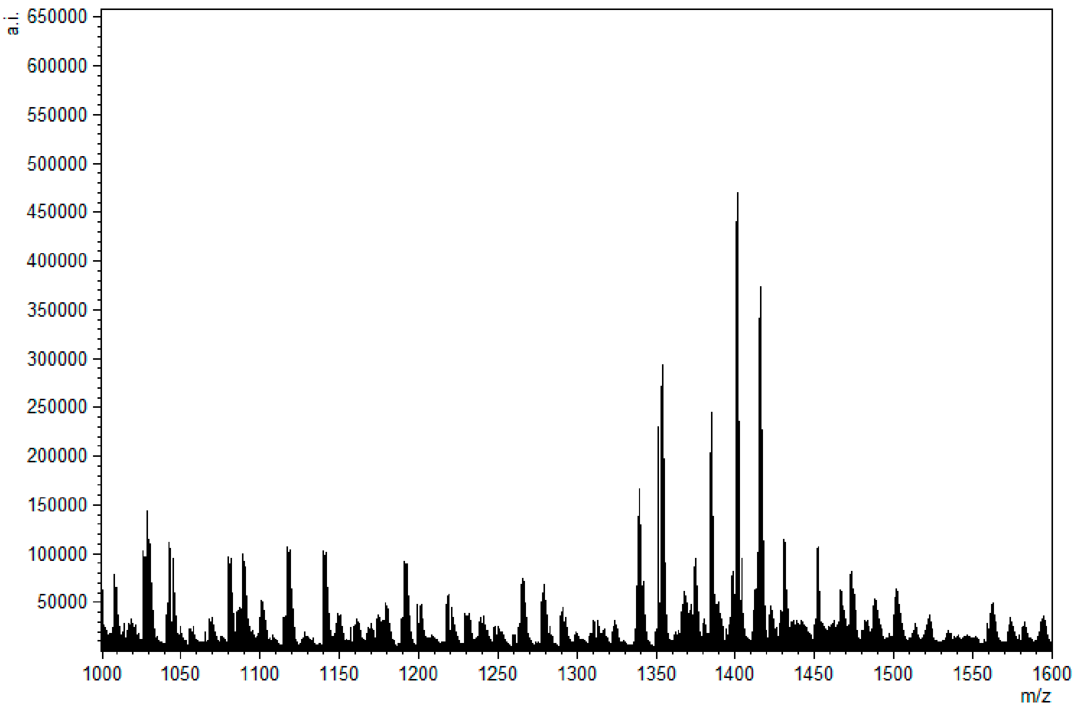

Figure S1. HR ESI-MS of compound **Ce6–C$_{60}$** (MeOH, negative mode, range: 1000-1600 m/z)

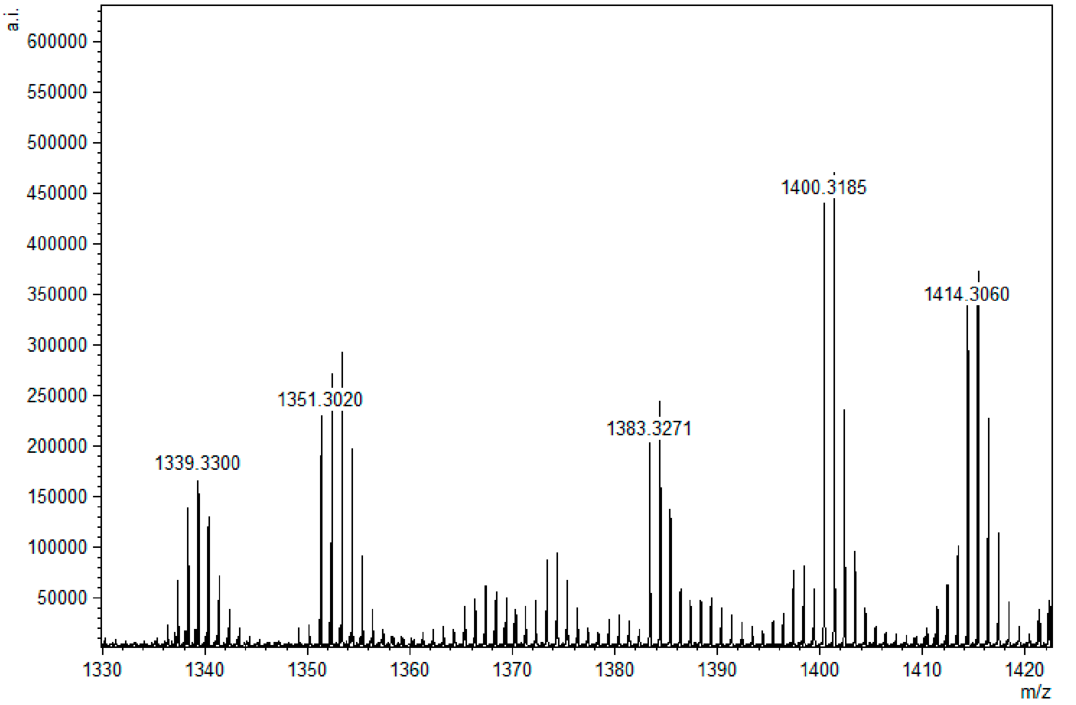

Figure S2. HR ESI-MS of compound **Ce6–C$_{60}$** (MeOH, negative mode, range: 1330-1420 m/z)

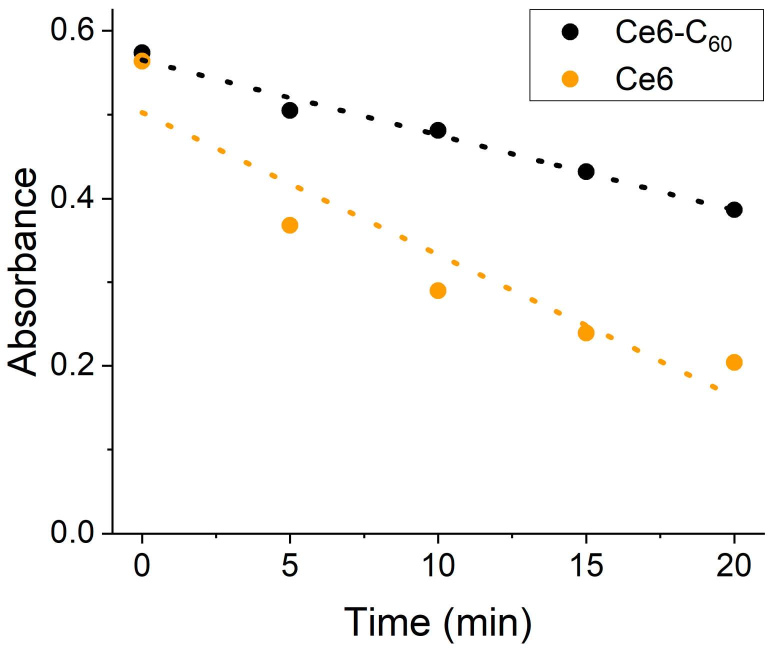

Figure S3. Absorbance at 401 nm of ABMDMA vs. irradiation time, under red light irradiation (irradiance 6.32 mW/cm^2), in the presence of Ce6–C_{60} (black dots) and Ce6 (gold dots) at 0.5 µM concentration. Curve fitting equations (represented as dotted lines) are used to calculate the quantum yield.

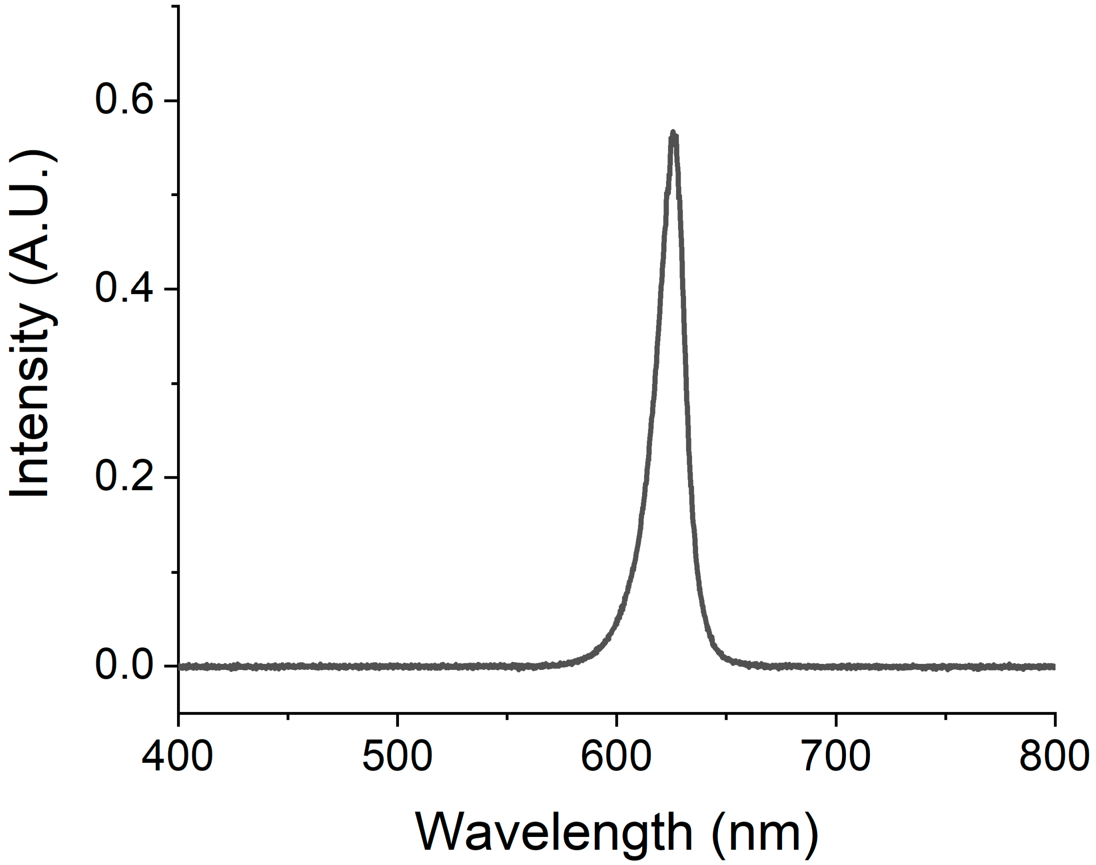

Figure S4. Spectral profile of the red light lamp (LED SMD 50W RGB)

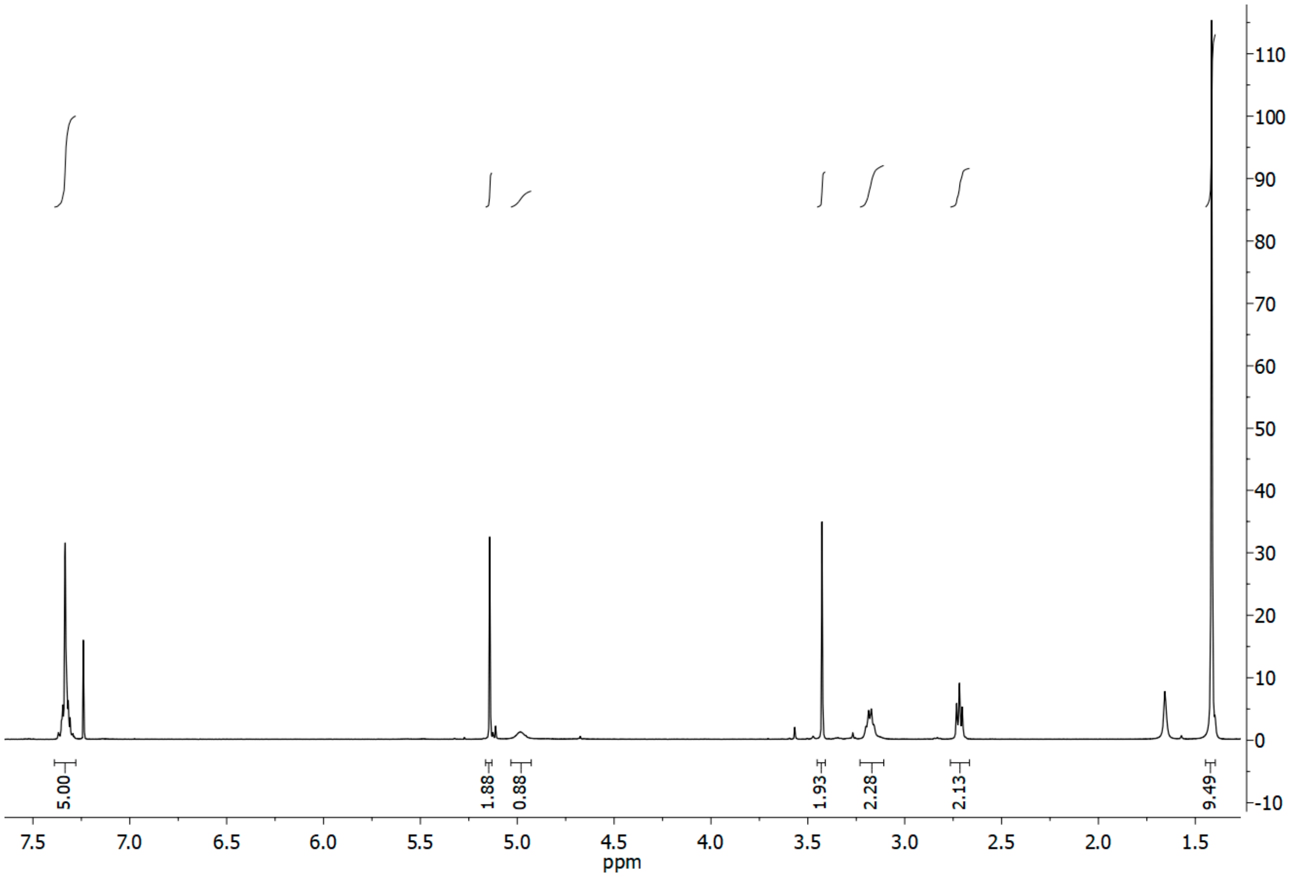

Figure S5. ^{1}H NMR of compound **2** (400 MHz, Chloroform-*d*)

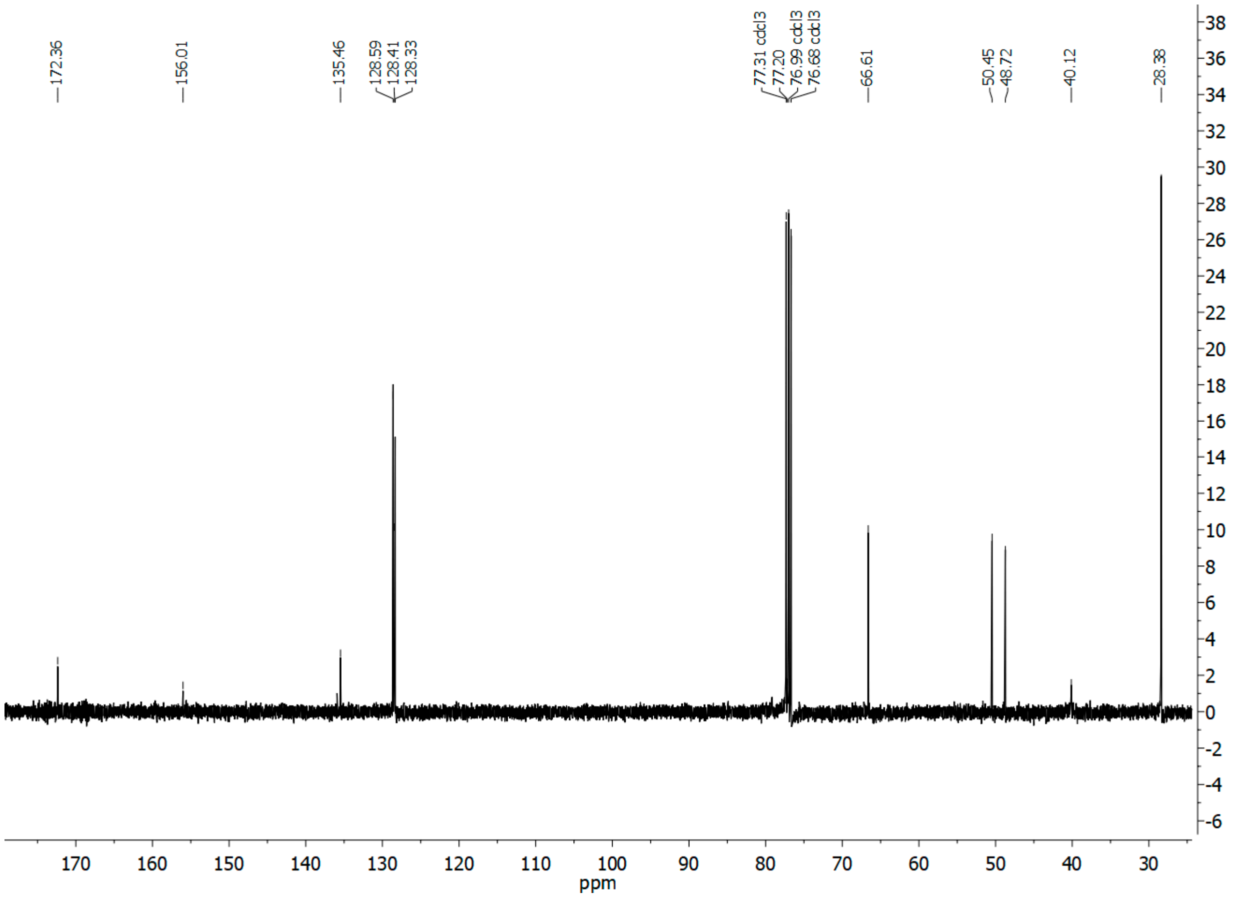

Figure S6. ^{13}C NMR of compound **2** (100 MHz, Chloroform-*d*)

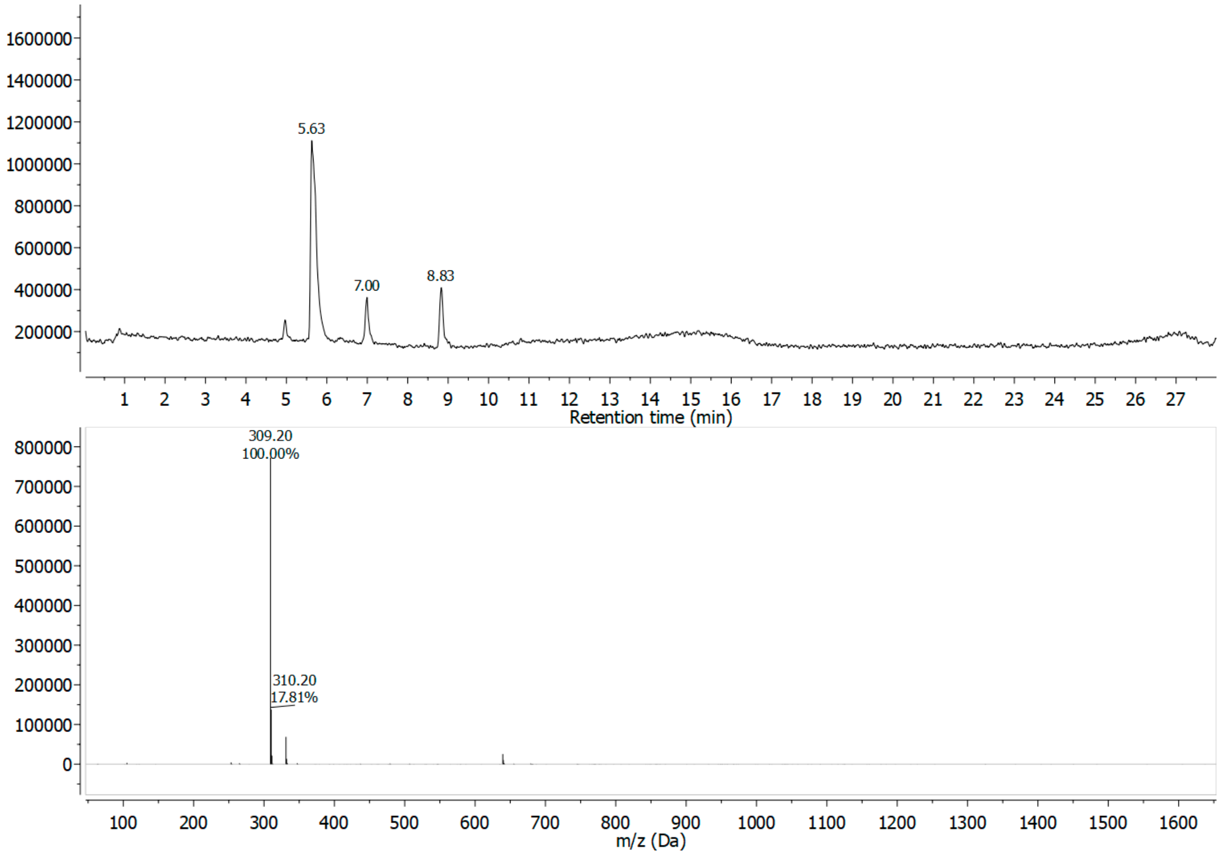

Figure S7. HPLC-MS of compound **2** (Acetonitrile)

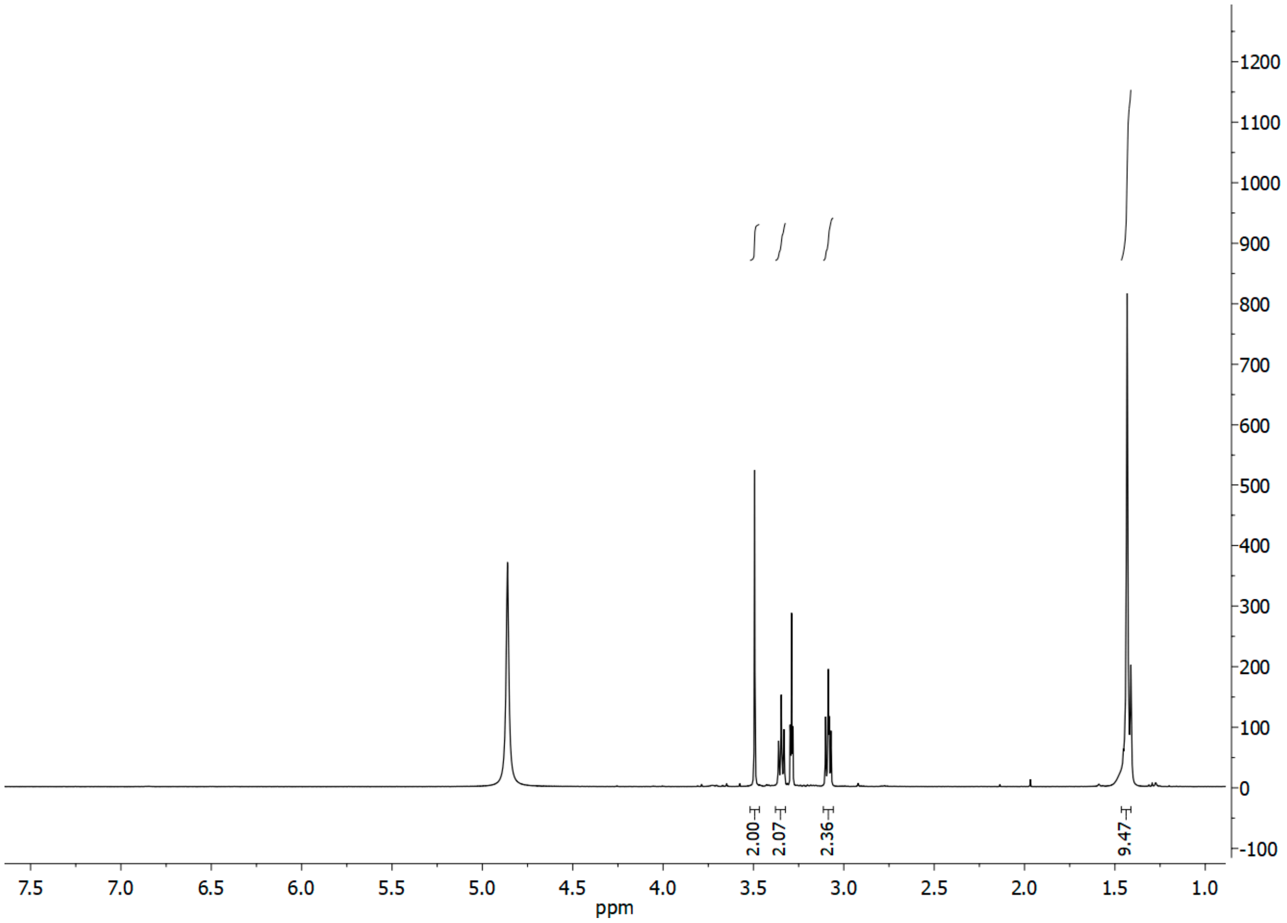

Figure S8. ^{1}H NMR of compound **3** (400 MHz, CD_3OD)

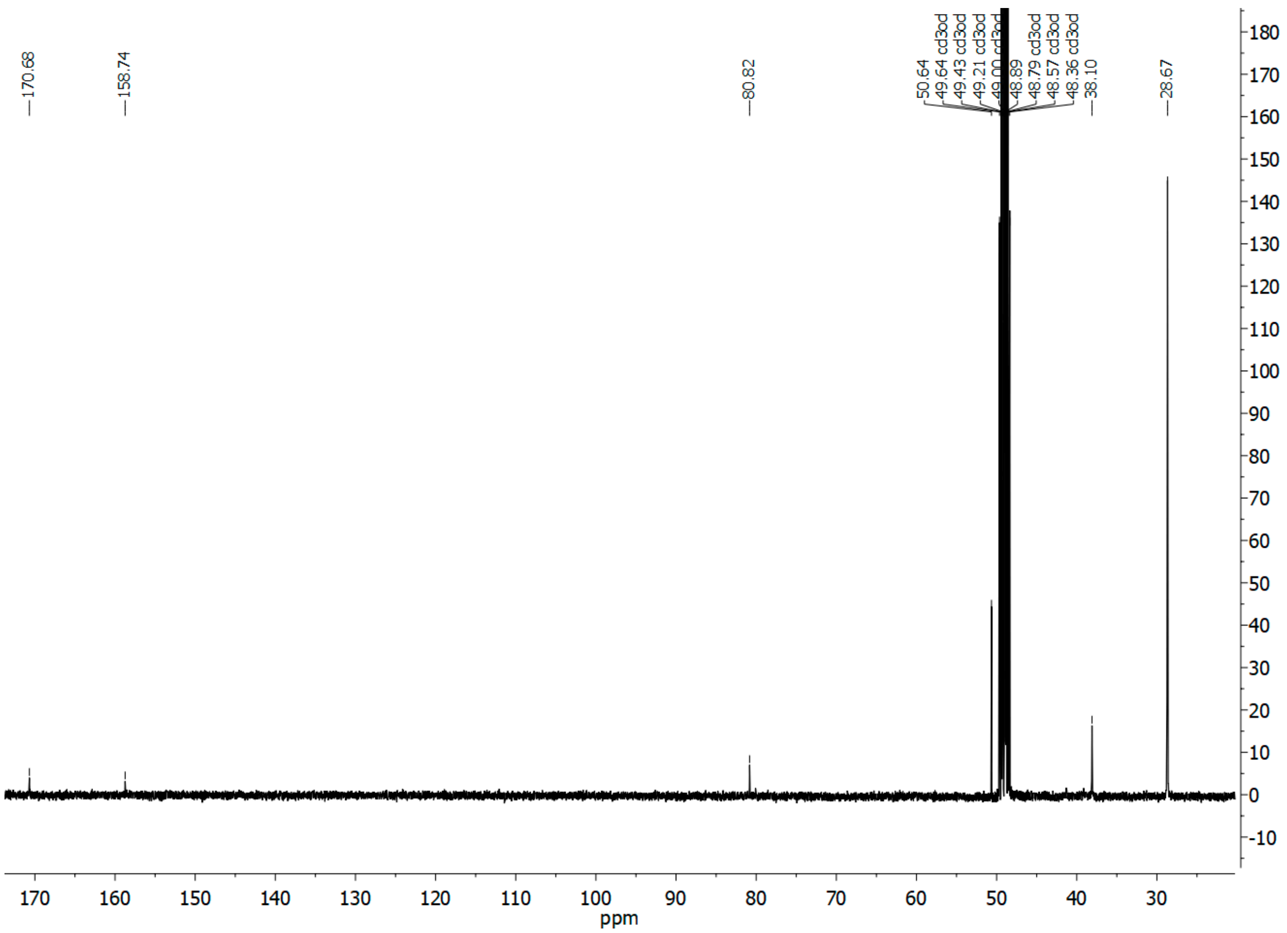

Figure S9. ^{13}C NMR of compound **3** (100 MHz, CD_3OD)

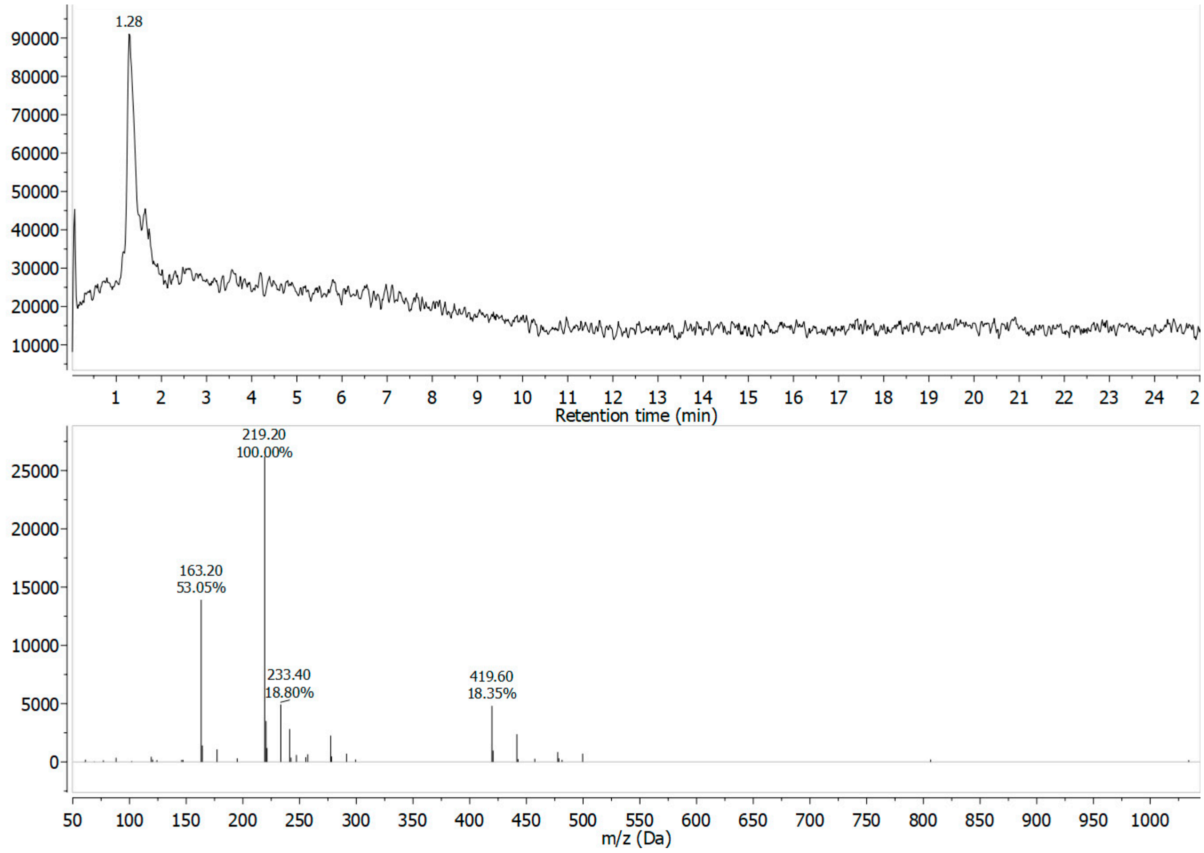

Figure S10. HPLC-MS of compound **3** (Acetonitrile)

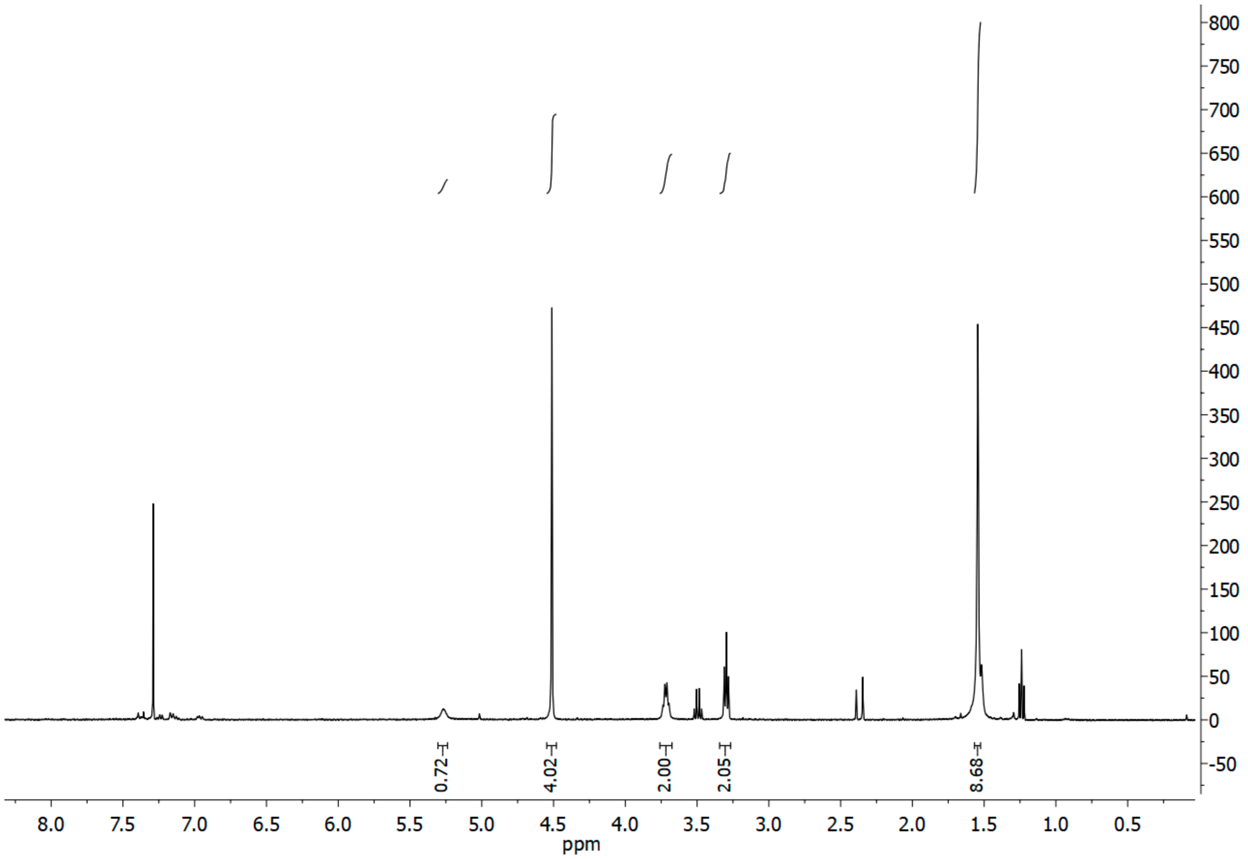

Figure S11. ^{1}H NMR of compound **4** (400 MHz, Chloroform-d:CS_2 1:1)

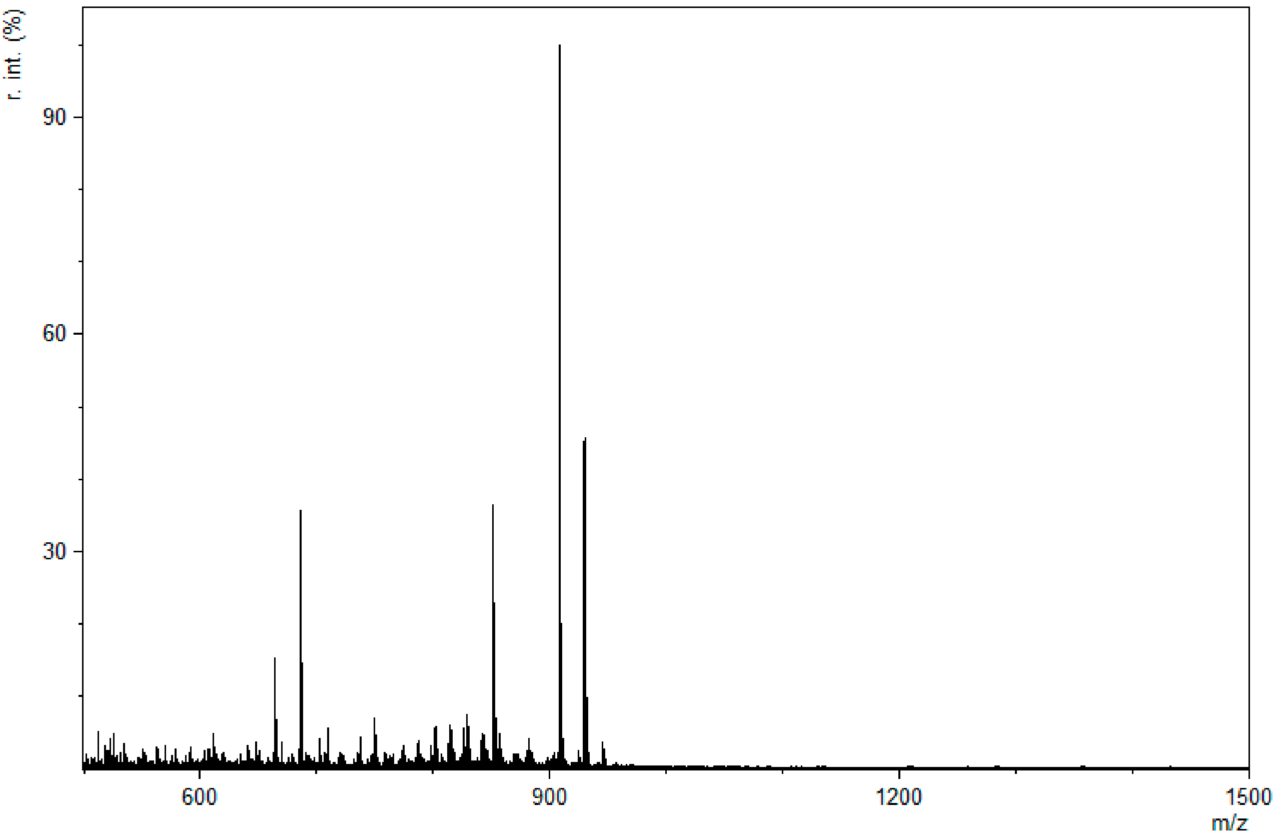

Figure S12. HR ESI-MS of compound **4** (MeOH, range: 500-1500 m/z)

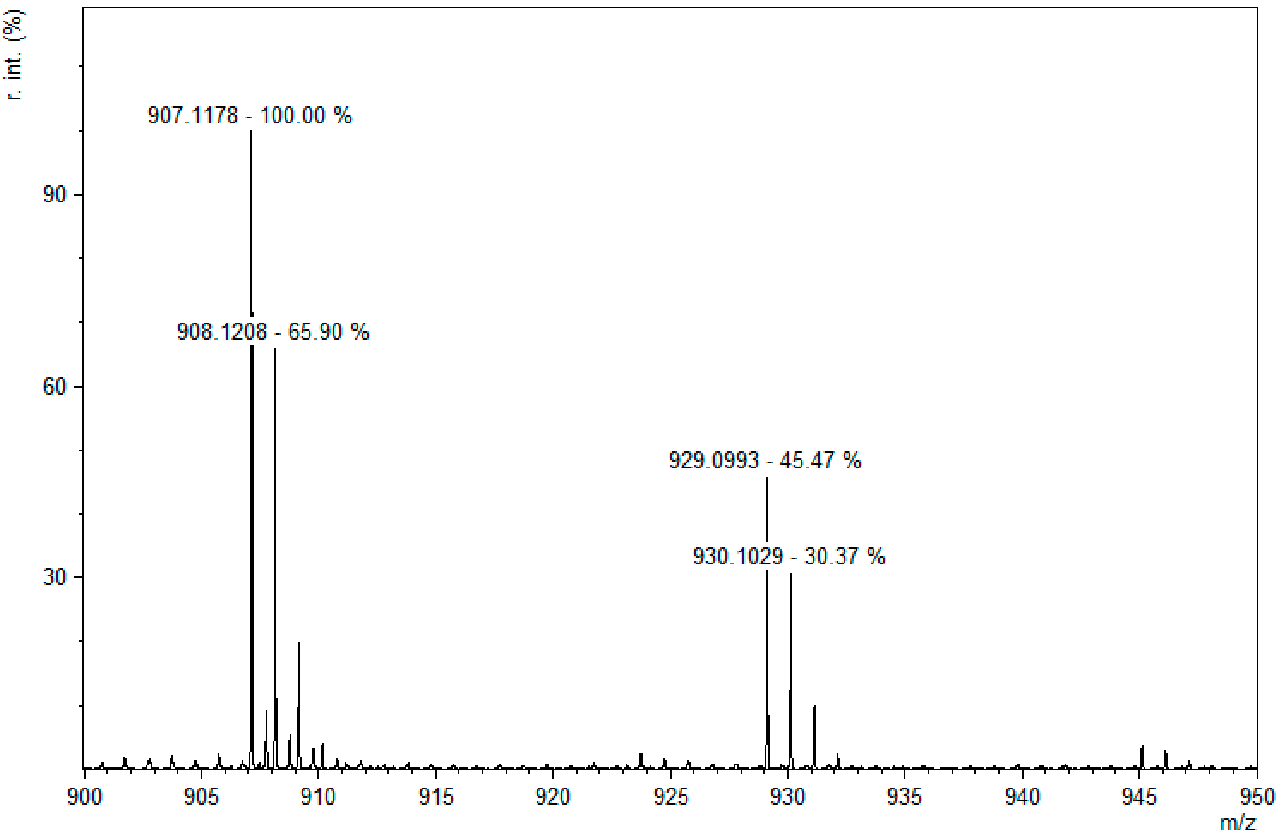

Figure S13. HR ESI-MS of compound **4** (MeOH, range: 900-950 m/z)

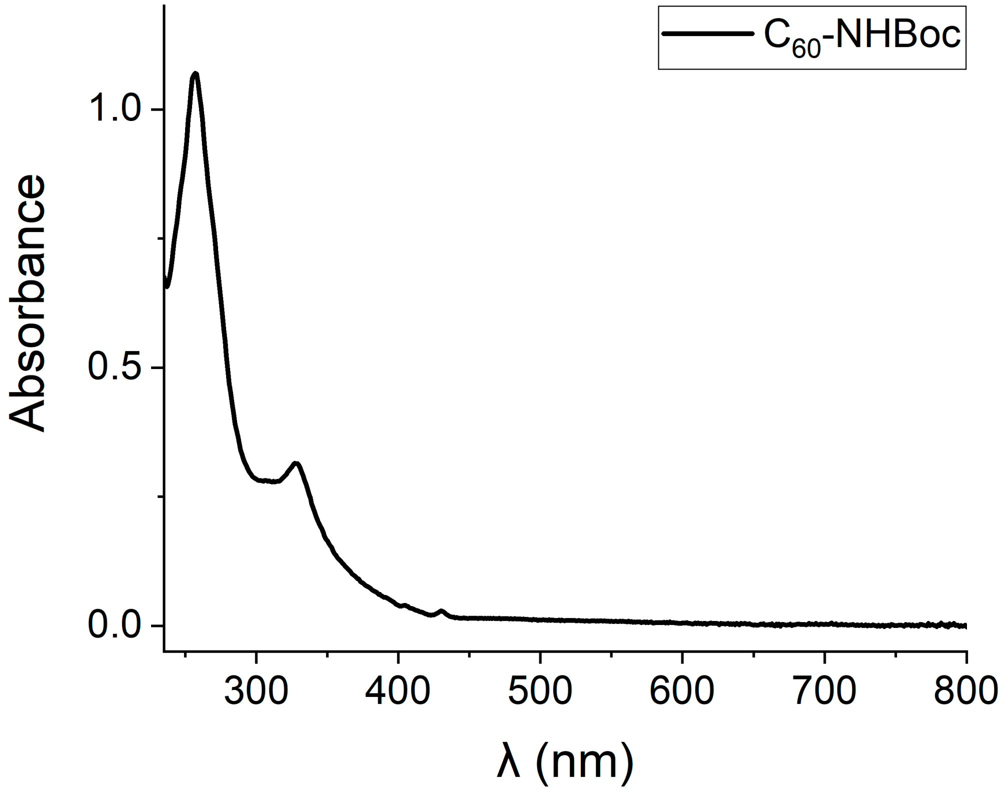

Figure S14. UV-Vis spectrum of compound **4** (Dichloromethane, 230-800 nm)

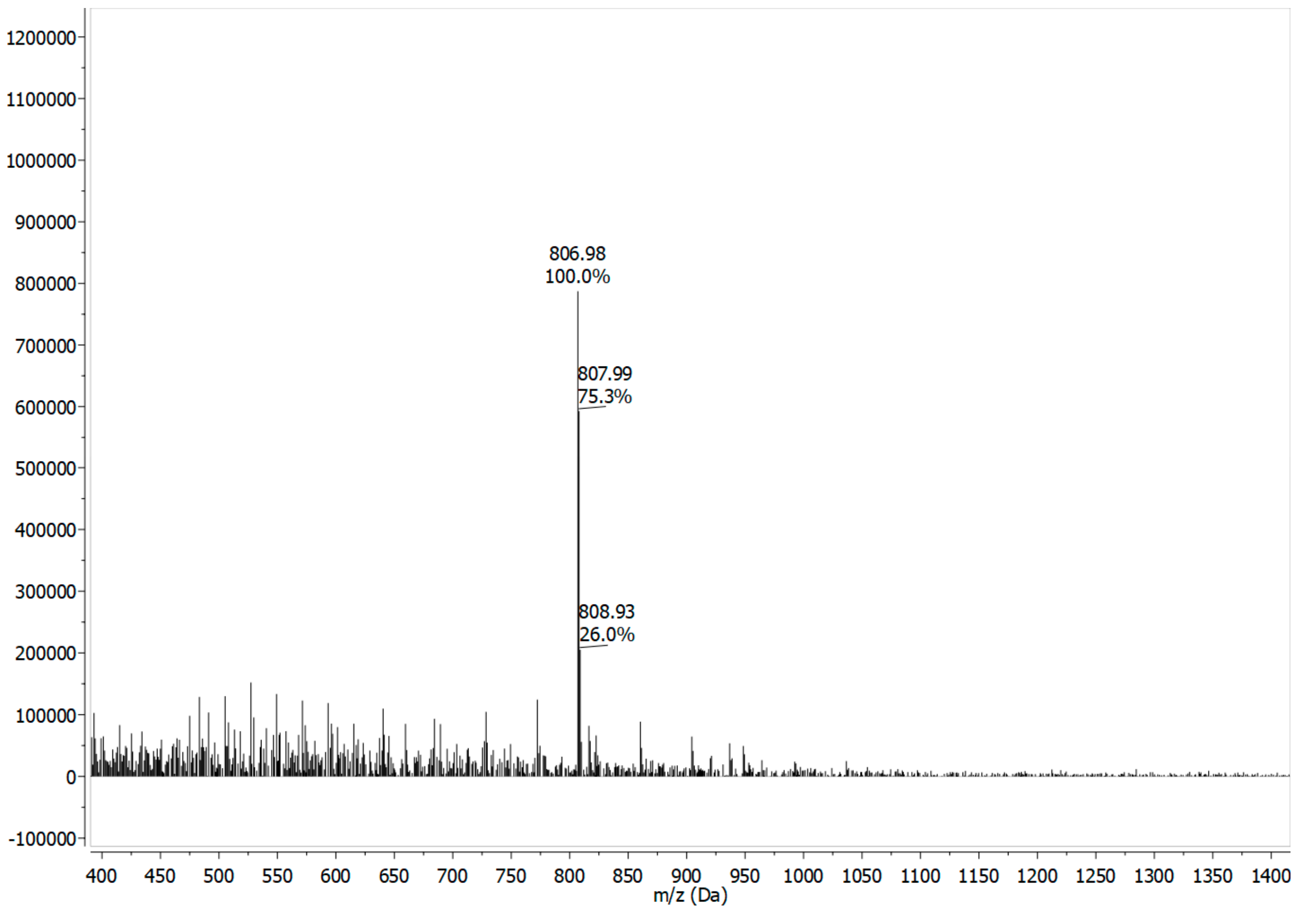

Figure S15. ESI-MS of compound **5** (MeOH, range: 400-1400 m/z)

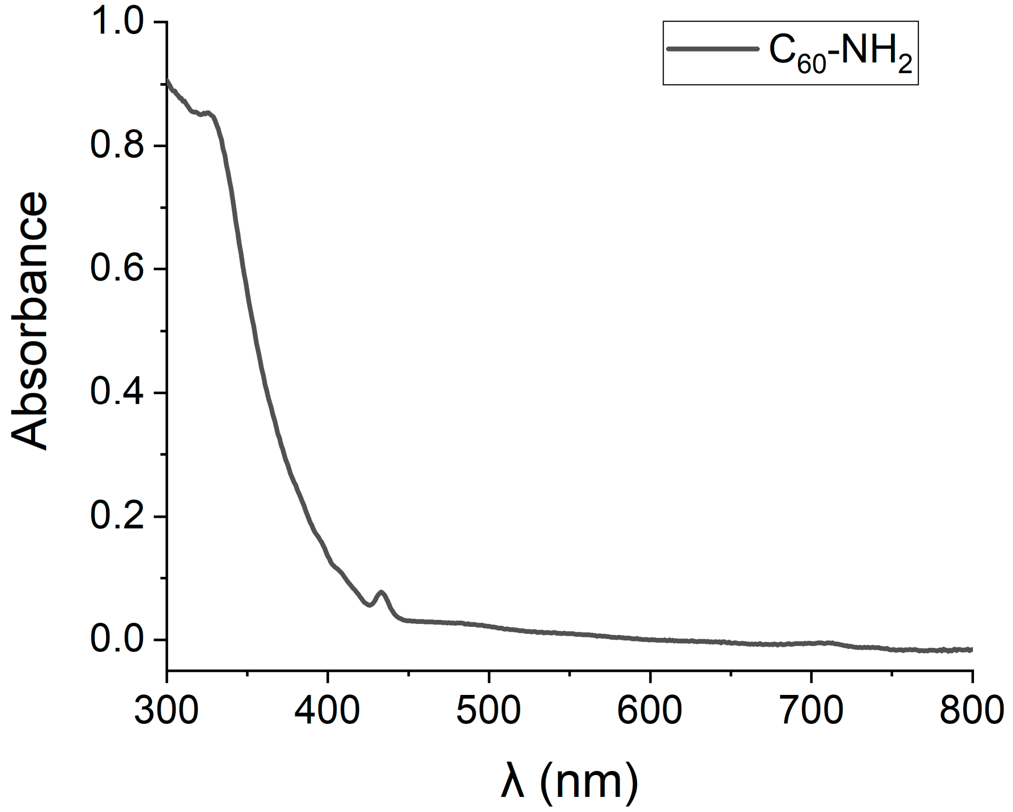

Figure S16. UV-Vis spectrum of compound **5** (DMSO, 300-800 nm).
